# Canadian recommendations for the treatment of glioblastoma multiforme

**DOI:** 10.3747/co.2007.119

**Published:** 2007-06

**Authors:** W.P. Mason, R. Del Maestro, D. Eisenstat, P. Forsyth, D. Fulton, N. Laperrière, D. Macdonald, J. Perry, B. Thiessen, for the Canadian GBM Recommendations Committee

**Keywords:** Brain tumour, glioblastoma, radiotherapy, chemotherapy, temozolomide

## Abstract

**Recommendation 1:**

Management of patients with glioblastoma multiforme (gbm) should be highly individualized and should take a multidisciplinary approach involving neuro-oncology, neurosurgery, radiation oncology, and pathology, to optimize treatment outcomes. Patients and caregivers should be kept informed of the progress of treatment at every stage.

**Recommendation 2:**

Sufficient tissue should be obtained during surgery for cytogenetic analysis and, whenever feasible, for tumour banking.

**Recommendation 3:**

Surgery is an integral part of the treatment plan, to establish a histopathologic diagnosis and to achieve safe, maximal, and feasible tumour resection, which may improve clinical signs and symptoms.

**Recommendation 4:**

The preoperative imaging modality of choice is magnetic resonance imaging (mri) with gadolinium as the contrast agent. Other imaging modalities, such as positron emission tomography with [^18^F]-fluoro-deoxy-d-glucose, may also be considered in selected cases. Postoperative imaging (mri or computed tomography) is recommended within 72 hours of surgery to evaluate the extent of resection.

**Recommendation 5:**

Postoperative external-beam radiotherapy is recommended as standard therapy for patients with gbm. The recommended dose is 60 Gy in 2-Gy fractions. The recommended clinical target volume should be identified with gadolinium-enhanced T1-weighted mri, with a margin in the order of 2–3 cm. Target volumes should be determined based on a postsurgical planning mri. A shorter course of radiation may be considered for older patients with poor performance status.

**Recommendation 6:**

During rt, temozolomide 75 mg/m^2^ should be administered concurrently for the full duration of radio-therapy, typically 42 days. Temozolomide should be given approximately 1 hour before radiation therapy, and at the same time on the days that no radiotherapy is scheduled.

**Recommendation 7:**

Adjuvant temozolomide 150 mg/m^2^, in a 5/28-day schedule, is recommended for cycle 1, followed by 5 cycles if well tolerated. Additional cycles may be considered in partial responders. The dose should be increased to 200 mg/m^2^ at cycle 2 if well tolerated. Weekly monitoring of blood count is advised during chemoradiation therapy in patients with a low white blood cell count. *Pneumocystis carinii* pneumonia has been reported, and prophylaxis should be considered.

**Recommendation 8:**

For patients with stable clinical symptoms during combined radiotherapy and temozolomide, completion of 3 cycles of adjuvant therapy is generally advised before a decision is made about whether to continue treatment, because pseudo-progression is a common phenomenon during this time. The recommended duration of therapy is 6 months. A longer duration may be considered in patients who show continuous improvement on therapy.

**Recommendation 9:**

Selected patients with recurrent gbm may be candidates for repeat resection when the situation appears favourable based on an assessment of individual patient factors such as medical history, functional status, and location of the tumour. Entry into a clinical trial is recommended for patients with recurrent disease.

**Recommendation 10:**

The optimal chemotherapeutic strategy for patients who progress following concurrent chemoradiation has not been determined. Therapeutic and clinical–molecular studies with quality of life outcomes are needed.

## 1. INTRODUCTION

Glioblastoma multiforme (gbm) is a World Health Organization grade iv astrocytoma and the most common and aggressive primary brain tumour 1. In North America, the estimated age-adjusted incidence of gbm is 3.0 per 100,000 population 1. It occurs more commonly in males (male:female ratio of approximately 3:2) and is typically diagnosed in patients in their sixth or seventh decade.

The preoperative imaging modality of choice is gadolinium-enhanced magnetic resonance imaging (mri). Although contrast-enhanced mri may indicate a discrete border, gbm tumours are characterized by extensive microvascular infiltration and rapid proliferation. Based on distinct pathogenetic features, at least two subtypes of gbm can be defined. Primary (*de novo*) glioblastoma is more common in older patients (mean age: 55 years) 2 and typically harbours overexpression or mutation of epidermal growth factor receptor, genetic losses on chromosome 10, p16 or p19 alterations, or loss of the tumour suppressor protein phosphatase and tensin homologue 3–6. Secondary glioblastoma develops more slowly from a lower-grade tumour and typically occurs in younger patients (≤45 years). Genetic alterations may include *TP53* mutation or overexpression of platelet-derived growth factor receptor α 7.

Although imaging techniques and multimodal treatment strategies have improved since the mid-1980s, little impact has been made on the ultimate prognosis of gbm. A population-based cohort study of all Ontario Cancer Registry cases of gbm identified between 1982 and 1994 found that the median survival of patients receiving radiotherapy (rt) and surgery was 11 months; median survival with surgery alone was 3 months 8. An analysis of the Alberta Cancer Registry database of gbm patients diagnosed between 1975 and 1991 reported that only 1.8% survived at least 3 years 9. A decade later, an analysis by the Glioma Outcomes Project of cases diagnosed between 1997 and 2001 reported a median survival of 40.9 weeks for newly-diagnosed patients with gbm 10. An analysis of the Surveillance, Epidemiology, and End Results database found no significant improvement in the gbm survival rate after the 1980s 1.

The principal reasons for poor outcome in gbm are the high rates of recurrence and of resistance to chemotherapy. Choucair *et al.* estimated that more than 90% of gliomas recur, typically at the site of the original tumour 11. Numerous chemotherapy regimens, administered either before rt or adjuvantly, have been investigated, but they have had little impact on patient outcomes 12–16. Prognosis is affected by the histologic features of the tumour, patient age, and performance status 17,18.

Standard treatment for gbm was significantly altered following the results of a large phase iii trial conducted by the European Organization for the Research and Treatment of Cancer (eortc) and the National Cancer Institute of Canada 19. The eortc ncic ce3 trial randomized 573 newly-diagnosed glioblastoma patients to rt alone (2 Gy, 5/7-day schedule for 6 weeks, 60 Gy total), or to rt in combination with the oral alkylating agent temozolomide. The temozolomide dose was 75 mg/m^2^ daily during rt, followed by adjuvant temozolomide 150–200 mg/m^2^ daily in a 5/28-day schedule for 6 cycles. With the rt–temozolomide combination, 2-year survival was 26.5% as compared with 10.4% with rt alone. Median survival was 12.1 months and 14.6 months respectively. As a result, concurrent rt and temozolomide, followed by 6 monthly cycles of adjuvant temozolomide, became the new standard of care for patients newly diagnosed with gbm.

However, numerous questions remain about how to identify patients who will be more likely to respond to treatment and how to optimize a multimodal approach to patient management. The recommendations that follow were developed by a multidisciplinary panel of Canadian neuro-oncologists, neurosurgeons, and radiation oncologists—based on level 1 evidence where possible—as a guide to optimizing the management of patients with gbm.

## 2. THE RECOMMENDATIONS

### 2.1 General Principles

#### Recommendation 1

Management of patients with gbm should be highly individualized and should take a multidisciplinary approach involving neuro-oncology, neurosurgery, radiation oncology, and pathology, to optimize treatment outcomes. The care path of gbm is complex and requires the cooperation and integration of services from multiple health care specialties and institutions so as to avoid unacceptable wait times.

All surgeries should be presented at a weekly brain tumour conference, with the neurosurgeon, radiation oncologist, and neuro-oncologist present. Ideally, for each case, the multidisciplinary team should review the patient’s clinical status, neuroimaging, and histopathologic findings to determine the optimal treatment approach.

It is recommended that the neurosurgeon inform the patient of the diagnosis. Patients and caregivers should be kept informed of the progress of treatment at every stage. Patients should receive a brain tumour information package to help them understand gbm and the treatment options, and to better inform their decision-making. Patient consent should be obtained for tumour banking.

### 2.2 Pathology

Specific, unique genetic changes are common in astrocytic tumours. An estimated one half of grades ii–iii infiltrating astrocytomas have detectable mutations in the *TP53* tumour suppressor gene 20. Loss of heterozygosity on chromosomes 1p and 19q is usually associated with oligodendroglioma, but sometimes occurs in oligoastrocytomas. Loss of heterozygosity is associated with increased sensitivity to pro-carbazine–lomustine–vincristine chemotherapy 21. In that regard, two eortc phase ii trials reported that first-or second-line temozolomide produced a high response rate in patients with recurrent or progressive oligoastrocytoma or oligodendroglioma 22,23; response was associated with 1p and 19q loss 24. Glioblastoma multiforme is more chemoresistant, and genetic markers do not appear to have comparable prognostic significance 25. Of particular importance, however, is O^6^-methylguanine dna methyltransferase (mgmt), a repair protein that removes methyl adducts and transfers them to an internal cysteine residue 26. Because the O^6^ position is one of the targets of alkylating chemotherapeutic agents, mgmt activity enhances tumour resistance by repairing cytoxic damage. Conversely, tumour sensitivity is enhanced if mgmt is silenced through hypermethylation of the CpG islands in the promoter region 27–29.

A number of studies have indicated that mgmt promoter methylation is predictive of a good response to alkylating agents such as 1,3-bis(2-chloroethyl)-1-nitrosourea (bcnu) and cyclophosphamide 30,31. Following the phase iii study of combined rt and temozolomide 19, Hegi *et al.* analyzed the mgmt promoter methylation status of 206 evaluable patients 32. In 92 tumours (44.7%), mgmt promoter methylation was detectable. Median survival was 18.2 months in patients with promoter methylation as compared with 12.2 months in those without methylation, and median survival was 21.7 months in rt–temozolomide-treated patients with promoter methylation. In the subgroup of patients with promoter methylation, 2-year survival was 46% in the rt–temozolomide group as compared with 22.7% in patients treated with rt alone (Table I). In the subgroup of patients without promoter methylation, median survival was only marginally superior with combined rt–temozol-omide than with rt alone (12.7 months vs. 11.8 months); 2-year survival was 13.8% as compared with <2% respectively.

Temozolomide is a recommended treatment for newly-diagnosed gbm and for recurrent high-grade gliomas. Although mgmt methylation status appears to be a prognostic factor for increased survival and possibly for better response to temozolomide, a prospective study is required before promoter hyper-methylation can be used as a guide to treatment decisions in gbm.

#### Recommendation 2

The molecular genetic determination of brain tumours is becoming increasingly important, enabling more accurate diagnosis and prognosis. Sufficient tissue should be obtained during surgery for cytogenetic analysis and, whenever feasible, for tumour banking. The preliminary pathology report should be available within 48 hours post surgery; the final report should be completed within 8 working days post surgery.

### 2.3 Surgery

#### Recommendation 3

Surgery is an integral part of the treatment plan, to establish a histopathologic diagnosis and to achieve safe maximal tumour resection, which may improve clinical signs and symptoms.

A gross total resection, if achievable, is advised in any patients with a primary or recurrent malignant glial tumour if the surgery can be performed without significant risk to the patient. Simpson *et al.* analysed data from 645 gbm patients in three prospective Radiation Therapy Oncology Group trials 33. Surgery consisted of total resection (19%), partial resection (64%), or biopsy only (17%). Median survival was 11.3 months for total resection, 10.4 months for partial resection, and 6.6 months for biopsy.

#### Recommendation 4

The preoperative imaging modality of choice is mri with gadolinium as the contrast agent. Other imaging modalities, such as positron emission tomography with [^18^F]-fluoro-deoxy-d-glucose, may also be considered in selected cases 34. Postoperative imaging (mri or computed tomography) is recommended within 72 hours of surgery to evaluate the extent of resection.

### 2.4 Radiotherapy

The use of adjuvant external-beam rt is well established in the postoperative treatment of gbm. A pooled analysis of six randomized trials by Cancer Care Ontario reported a significant survival benefit favouring postoperative rt as compared with no rt (risk ratio: 0.81) 35,36. Overall, median survival is approximately 36–48 weeks with adjuvant rt as compared with 14–22 weeks with surgery alone 37–39.

External-beam rt is generally administered over 5–6 weeks, delivering a total dose of 50–60 Gy in 1.8- to 2.0-Gy fractions 40. Doses above 60 Gy and boost rt do not appear to influence survival 40,41.

Alternative forms of fractionation have been investigated. Accelerated fractionation delivers standard fraction sizes more frequently (for example, 2 or 3 times daily) to reduce the overall treatment time. Several studies have reported no increased survival, although no increased toxicity was found 42–44. This approach may be an option for selected patients (such as the elderly), but additional study is needed.

Hyperfractionation, which delivers a higher total radiation dose in a larger number of smaller fractions, showed no improvement in time to tumour progression or survival 45,46.

Radiotherapy should be initiated within 4 weeks of surgery.

#### Recommendation 5

Postoperative external-beam rt is recommended as standard therapy for patients with gbm. The recommended dose is 60 Gy in 2-Gy fractions 35,36. The recommended clinical target volume should be identified with gadolinium-enhanced T1-weighted mri, with a margin in the order of 2–3 cm, given that most recurrences will occur within a few centimetres of the tumour mass 47,48. Target volumes should be determined based on a postsurgi-cal planning mri. A shorter course of radiation may be considered for older patients with poor performance status 49,50.

### 2.5 Chemotherapy

Glioblastoma multiforme has been viewed as a chemoresistant tumour, and the nitrosoureas, the traditional mainstays of treatment, have had modest efficacy, but are associated with significant toxicity 39. Most studies were reported decades ago, but a recent phase ii trial evaluated bcnu 80 mg/m^2^ on days 1–3 every 8 weeks (maximum 6 cycles) in 40 patients with recurrent gbm who had undergone surgery and RT^51^. The median time to progression was 13 weeks; the 6-month progression-free survival (pfs) was 17.5%. Significant side effects included reversible hematologic toxicities and chronic hepatic and pulmonary toxicity.

#### Recommendation 6

During rt, temozolomide 75 mg/m^2^ should be administered concurrently for 42 days 35. Temozolomide should be given approximately 1 hour before rt, and at the same time on the days when no rt is scheduled (weekends).

Whether the clinical benefit of this combination is attributable in part to the radiosensitizing effects of temozolomide is unclear. To date, four *in vitro* studies have suggested a radiosensitizing effect with tem-ozolomide for some cancer cell lines 52–55, but additional research is needed.

#### Recommendation 7

Adjuvant temozolomide 150 mg/m^2^, in 5/28-day schedule, is recommended for cycle 1, followed by 5 cycles if well tolerated. Additional cycles may be considered in partial responders or in those with continuing radiologic improvement. The dose should be increased to 200 mg/m^2^ at cycle 2 if well tolerated. Weekly monitoring of blood count is advised during chemoradiation therapy in patients with a low white blood cell count. *Pneumocystis carinii* pneumonia has been reported, and prophylaxis should be considered 56.

#### Recommendation 8

For patients with stable clinical symptoms during rt–temozolomide, completion of 3 cycles of adjuvant therapy is generally advised before a decision is made about whether to continue treatment. In the first few weeks or months following completion of rt, mri is not reliable to assess true progression. Evidence of progression outside the rt field is indicative of true progression. A longer duration may be considered in patients who show continuous improvement on therapy.

### 2.6 Recurrent GBM

#### Recommendation 9

Selected patients with recurrent gbm may be candidates for repeat resection when the situation appears favourable based on an assessment of individual patient factors such as medical history, functional status, and location of the tumour 57,58. Entry into a clinical trial is recommended for patients with recurrent disease.

#### Recommendation 10

The optimal chemotherapeutic strategy for patients who progress following concurrent chemoradiation has not been determined. Therapeutic and clinical-molecular studies with quality of life outcomes are needed 35.

For patients not receiving chemotherapy at the time of progression, re-challenge with temozolomide to deplete mgmt might be attempted, but clinical data on this strategy are lacking.

Some preliminary data suggest that novel dose-intense schedules may provide some benefit. Khan *et al.* reported a 6-month pfs of 19% with temozolomide 75 mg/m^2^ in a 42/70-day schedule 59. In a small phase ii study by Wick *et al.*, the 6-month pfs was 48% with temozolomide 150 mg/m^2^ administered in a 7-day on / 7-day off schedule 60. Although the findings are promising, additional phase ii studies are required before the foregoing dosing regimens can be recommended.

A number of chemotherapeutic agents, including nitrosoureas, carboplatin, etoposide, irinotecan, and imatinib 61–64, have been used as salvage therapy either alone or in combination. Additional trials with a variety of agents are underway, but preliminary results from single-agent studies have been disappointing. Table II summarizes phase ii studies in gbm.

For patients who progress on temozolomide, combination therapy may be possible; several recent trials have evaluated various temozolomide combinations (Table II). For example, the efficacy of bolus temozolomide 130 mg/m^2^ followed by 70 mg/m^2^ every 12 hours for 5 days, plus cisplatin 75 mg/m^2^, was evaluated in 50 patients with recurrent gbm 65. Among the 49 evaluable patients, 1 patient achieved a complete response, and 9 achieved partial responses. The 6-month pfs was 34%; the 12-month pfs was 4%. Overall survival was 11.2 months. The most common grade 3–4 toxicity was granulocytopenia, which occurred in 8% of cycles.

## 3. CONCLUSIONS

Surgery followed by rt still represents the primary approach to the treatment of gbm. The addition of temozolomide chemotherapy to the standard of care has significantly increased the proportion of patients who survive more than 2 years. However, additional progress still needs to be made, because almost one half of gbm patients will not survive the first year after surgery. Additional research is needed to build on recent clinical gains and to focus on new drug combinations or therapies that could potentially further improve outcomes in patients with gbm.

## Figures and Tables

**TABLE I:** Effect of methylation status of methylguanine DNA methyltransferase (mgmt) promoter on progression-free survival (pfs) and overall survival (os) in patients receiving radiotherapy plus temozolomide (tmz) versus radiotherapy (RT) alone^a^

*Clinical endpoint*	*TMZ + RT (*n*=106)*	*RT (*n*=100)*
Methylated MGMT (*n*)	46	46
6-Month PFS	47.8	68.9
2-Year OS	22.7	46.0
Unmethylated MGMT (*n*)	54	60
6-Month PFS	35.2	40.0
2-Year OS	<2	13.8

a Adapted from reference 32.

**TABLE II:** Phase ii studies in recurrent glioblastoma multiforme

*Reference*	*Regimen*	*Patients (*n*)*	*6-Monthpfs(%)*
Yung *et al.*^65^	tmz 150–200 mg/m^2^ daily, 5/28-day schedule vs. pcb 125–150 mg/m^2^, 28/56-day schedule	225	21 8
Groves *et al.*^66^	tmz 150–200 mg/m^2^, 5/28-day schedule + marimastat 50 mg, days 8–28 × 2 cycles	44	39
Jaeckle *et al.*^67^	tmz 150–200 mg/m^2^, 5/28-day schedule + *cis-*retinoic acid 100 mg/m^2^, 21/28-day schedule	40	32
Brandes *et al.*^68^	tmz 130 mg/m^2^ bolus, tmz 70 mg/m^2^ every 12 hours × 5 days + cisplatin 75 mg/m^2^	50	34
Brandes *et al.*^51^	bcnu 80 mg/m^2^, days 1–3 every 8 weeks × 6 cycles maximum	40	17.5
Brandes *et al.*^69^	pcb 100 mg/m^2^ × 30 days + tamoxifen 100 mg daily	51	na
Kappelle *et al.*^70^	pcv	63	29
Fine *et al.*^71^	bcnu 200 mg/m^2^, day 1 of every 6-week cycle + thalidomide 800 mg daily (maximum 1200 mg)	38	27
Brandes *et al.*^72^	bcnu 100 mg/m^2^ on day 1 + irinotecan 175 mg/m^2^ weekly × 4 weeks in every 6 weeks (maximum 8 cycles)	42	30
Pipas *et al.*^73^	Paclitaxel 175 mg/m^2^ day 1 + topotecan 1.0 mg/m^2^ days 1–5	20	na^a^
Rich *et al.*^74^	Gefitinib 500 mg daily; dose escalation to 750–1000 mg	53	13
See *et al.*^75^	*cis-*Retinoic acid 100 mg/m^2^ daily, 21/28-day schedule	85	19
Chang *et al.*^76^	Temsirolimus (mTor inhibitor) 250 mg weekly	43	2.3

a Trial suspended because of significant hemotoxicity.

pfs = progression-free survival; tmz = temozolomide; pcb = procarbazine; bcnu = 1,3-bis(2-chloroethyl)-1-nitrosourea; na = not available; pcv = procarbazine–lomustine–vincristine.
