# ERRATUM

**Published:** 2007-10

An incorrect table was submitted earlier this year as part of the article “Canadian recommendations for the treatment of glioblastoma multiforme” ( Mason WP, Del Maestro R, Eisenstat D, *et al. Curr Oncol* 2007;14(3):110–17). The corrected version follows:

## Figures and Tables

**TABLE I:** Effect of methylation status of methylguanine dna methyltransferase (mgmt) promoter on progression-free survival (pfs) and overall survival (os) in patients receiving radiotherapy plus temozolomide (tmz) versus radiotherapy (rt) alone^a^

Clinical endpoint	tmz + rt (*n* = 106)	rt (*n* = 100)
Methylated mgmt (*n*)	46	46
6-Month pfs	68.9	47.8
2-Year os	46.0	22.7
Unmethylated mgmt (*n*)	60	54
6-Month pfs	40.0	35.2
2-Year os	13.8	<2

a Adapted from Hegi ME, Diserens AC, Gorlia T, *et al.* mgmt gene silencing and benefit from temozolomide in glioblastoma. *N Engl J Med* 2005;352:997–1003.

